# Statin prescription disparities in patients with breast cancer and diabetes for primary cardiovascular disease prevention

**DOI:** 10.3389/fonc.2024.1483918

**Published:** 2024-11-06

**Authors:** Alicia B. Yang, Grace Mhango, Chung Yin Kong, Jenny J. Lin, Juan P. Wisnivesky, Amanda Leiter

**Affiliations:** ^1^ Division of Endocrinology, Diabetes, and Bone Diseases, Icahn School of Medicine at Mount Sinai, New York, NY, United States; ^2^ Division of General Internal Medicine Department of Medicine, Icahn School of Medicine at Mount Sinai, New York, NY, United States

**Keywords:** breast cancer, diabetes, statin, cardiovascular prevention, disparities (health racial), cancer survivor

## Abstract

**Background:**

This brief report examines statin prescription trends for primary cardiovascular disease (CVD) prevention in breast cancer (BC) survivors with diabetes, a large population at particularly high CVD risk.

**Methods:**

A population-based, retrospective cohort study was conducted using Surveillance, Epidemiology, and End Results (SEER) cancer registry data linked to Medicare claims. We identified women with preexisting diabetes who were diagnosed with stage 0–III primary BC between 2008 and 2017 without preexisting CVD. We assessed statin prescription rates over time and also examined differences in statin prescription rates according to patient sociodemographic characteristics. Using a multivariate logistic regression adjusted for sociodemographic and clinical variables, independent predictors of statin prescription were identified.

**Results:**

Of 8,423 BC patients with diabetes without preexisting CVD, 5,698 (68%) had a statin prescription. Statin prescriptions increased over time (BC diagnosis year 2008–2009: 65%, 2010–2011: 67%, 2012–2013: 66%, 2014–2015: 69%, 2016–2017: 70%; *p* = 0.01) and differed by age (66–69: 66%, 70–74: 70%, 75–79: 69%, ≥80: 65%; *p* < 0.01) and race (White: 68%, Black: 62%, Latina: 66%, Other: 72%; *p* < 0.01). In a multivariate analysis, race (Black vs. White: OR 0.80, 95% CI: 0.68–0.95) remained a predictor of statin prescription.

**Conclusion:**

In older early-stage BC survivors, statin prescriptions increased over time and varied by age, race, and BC stage. These findings can potentially inform strategies to improve guideline-concordant statin prescriptions in a group at high risk for CVD and reduce disparities.

## Introduction

Patients with breast cancer (BC) and diabetes, a common comorbidity in BC with a prevalence of ~20%, are at particularly high risk of mortality and morbidity from cardiovascular disease (CVD) (1, 2). With advancements in BC screening and treatment, CVD is emerging as a leading cause of death in older early-stage BC survivors, the largest group of cancer survivors in the United States (>4 million) (1). BC survivors are at high CVD risk due to a high prevalence of CVD risk factors and exposure to cardiotoxic cancer treatments, including chemotherapy and radiation (3). Diabetes increases CVD mortality in this population even further (4). The prevalence of diabetes in BC survivors is increasing and disproportionately affects Black and Latina BC survivors, who also experience higher rates of CVD mortality after BC diagnosis (5–7).

Statins decrease CVD mortality in patients with both BC and diabetes and are important for survivorship care in this high-risk group (3, 8). In 2013, the American College of Cardiology broadened its guidelines to recommend statins for primary CVD prevention for almost all patients with diabetes ages 40–75 years, making >85% of patients with diabetes statin-eligible (8). However, little is known about statin prescription patterns in older BC survivors with diabetes since this paradigm change. As knowledge of statin prescription trends in BC survivors with diabetes is important for identifying care gaps and improving care in this population, we conducted a population-based, retrospective cohort study using the Surveillance, Epidemiology, and End Results (SEER) cancer registry linked to Medicare claims.

## Methods

We identified women diagnosed with stage 0–III primary BC (according to the American Joint Committee on Cancer criteria version 7) between 2008 and 2017, aged 66 and older, who had preexisting diabetes, had Medicare prescription data, had no preexisting CVD (myocardial infarction, congestive heart failure, peripheral vascular disease, and cerebrovascular disease), and were alive 1 year after BC diagnosis. Diagnosis of diabetes was defined by validated claims-based International Classification of Diseases (ICD) codes (9). Patient characteristics collected from SEER-Medicare included age at BC diagnosis, race/ethnicity, income (based on median income level from census tract or zip code), marital status, comorbidity burden, and claims for home health aide use (as a proxy for functional status). Race and marital status were obtained for the SEER registry through medical records review and are self-reported. Hispanic ethnicity was identified through algorithms incorporating birthplace and surname (10). Comorbidity burden was assessed by the modified Charlson comorbidity index (CCI) (excluding diabetes and cancer). CCI is a validated claims-based algorithm to assess comorbidity burden and is correlated with higher mortality risk (11, 12). Hyperlipidemia and hypertension were identified by claims codes validated in previous studies (13–15). Statin prescription was defined as at least one statin pharmacy claim within 1 year after BC diagnosis.

The chi-square test was used to compare demographic and clinical characteristics between patients who were and were not prescribed a statin. We used multiple logistic regression to determine independent predictors of statin prescription (a binary variable), as this method allowed us to account for variable confounding. We adjusted for variables known to influence statin prescriptions in previous literature, including age, race, income, diagnosis year, BC stage, CCI, hypertension, hyperlipidemia, BC stage, and diagnosis year (16, 17). *p*-values <0.05 were considered statistically significant. Analyses were conducted using SAS (version 9.4; SAS Institute, Inc., Cary, North Carolina, USA). The study was approved by Mount Sinai’s Institutional Review Board (#2201132).

## Results

Of 48,273 women older than 66 years with stage 0–III primary BC diagnosed between 2008 and 2017 with Medicare prescription claims, 11,874 had preexisting diabetes (25%). After excluding 2,957 patients with preexisting CVD and 494 who died less than 1 year after BC diagnosis, 8,423 patients were eligible for analysis, of which 5,698 (68%) had a statin prescription (**Table 1**). Statin prescription differed by age (66–69 years: 66%, 70–74: 70%, 75–79: 69%, ≥80: 65%; *p* < 0.01) and race (White: 68%, Black: 62%, Latina: 66%, Other: 72%; *p* < 0.01), did not differ by comorbidity burden (CCI 0: 68%, 1–2: 67%, ≥3: 67%; *p* = 0.87), and decreased with higher BC stage (0: 73%, I: 70%, II: 65%, III: 63%; *p* < 0.01). Income and marital status did not differ significantly by statin prescription status (*p* > 0.05 for all comparisons). BC patients with hyperlipidemia (76% vs. 51% for no hyperlipidemia; *p* < 0.01) and hypertension (69% vs. 62% for no hypertension; *p* < 0.01) were more likely to be prescribed statins. Patients who received hormone therapy (69% vs. 65% for no hormone therapy; *p* < 0.01), radiation (69% vs. 65% for no radiation; *p* < 0.01), and surgery (68% vs. 63% for no surgery; *p* = 0.04) were also more likely to receive a statin prescription. However, patients treated with chemotherapy (65% vs. 68% for no chemotherapy; *p* < 0.01) were less likely to be prescribed a statin.

**Table 1:** Study cohort characteristics by statin prescription.

Characteristic	Total (*N* = 8,423), *N*	Statin prescription (*N* = 5,698), *N* (%)	*p*-value*
Sociodemographic characteristics			
Age, *N* (%)			<0.01
66–69 years	1,926	1,268 (66%)	
70–74 years	2,644	1,855 (70%)	
75–79 years	1,927	1,327 (69%)	
≥80 years	1,924	1,248 (65%)	
Race, *N* (%)			<0.01
White	6,158	4,195 (68%)	
Black	858	531 (62%)	
Hispanic/Latina	647	425 (66%)	
Other	712	511 (72%)	
Income quintile, *N* (%)			0.06
First	1,547	1,016 (66%)	
Second	1,538	1,018 (66%)	
Third	1,606	1,072 (67%)	
Fourth	1,672	1,170 (70%)	
Fifth	1,935	1,333 (69%)	
Marital status, *N* (%)			0.49
Not married	4,491	3,031 (67%)	
Married	3,483	2,376 (68%)	
Clinical characteristics at diagnosis			
Home health aide, *N* (%)			0.40
No home health aide	7,812	5,294 (68%)	
Has home health aide	611	404 (66%)	
Hyperlipidemia, *N* (%)			<0.01
No	2,791	1,419 (51%)	
Yes	5,632	4,279 (76%)	
Hypertension, *N* (%)			<0.01
No	1,784	1,098 (62%)	
Yes	6,639	4,600 (69%)	
Modified Charlson comorbidity score, *N* (%)			0.87
0	6,042	4,097 (68%)	
1–2	2,143	1,442 (67%)	
>2	238	159 (67%)	
Tumor characteristics			
Year of diagnosis			0.01
2008–2009	1,289	838 (65%)	
2010–2011	1,383	927 (67%)	
2012–2013	1,589	1,049 (66%)	
2014–2015	1,976	1,357 (69%)	
2016–2017	2,186	1,527 (70%)	
Stage			<0.01
0	350	256 (73%)	
I	4,822	3,352 (70%)	
II	2,480	1,603 (65%)	
III	771	487 (63%)	
Cancer treatment			
Hormone therapy			<0.01
No	2,506	1,633 (65%)	
Yes	5,917	4,065 (69%)	
Radiation			<0.01
No	3,696	2,415 (65%)	
Yes	4,727	3,283 (69%)	
Surgery			0.04
No	358	225 (63%)	
Yes	8,019	5,447 (68%)	
Chemotherapy			<0.01
No	6,621	4,526 (68%)	
Yes	1,802	1,172 (65%)	

*Chi-square test.

**Figure 1:**
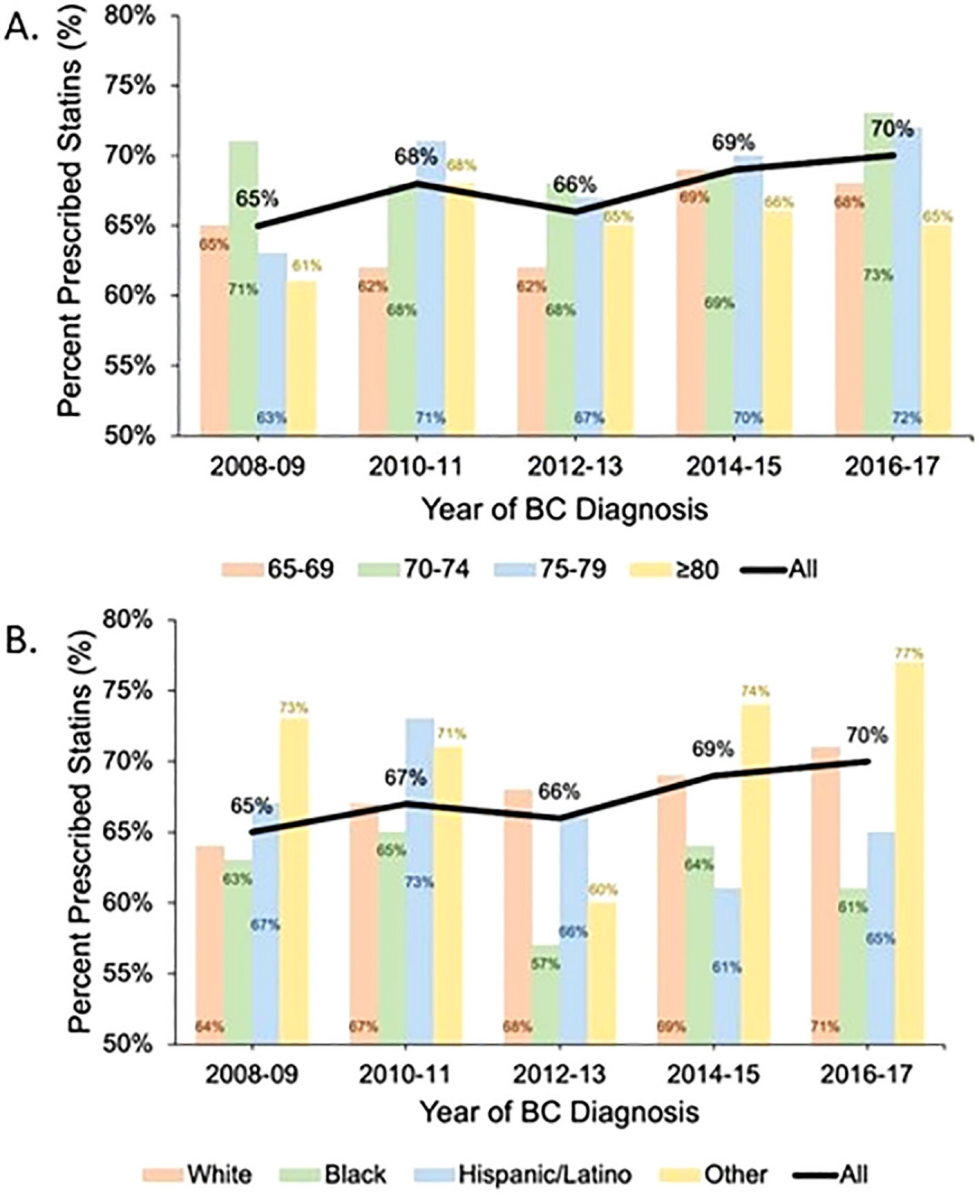
Statin prescription trends over time by age category and race: The black line shows the trend in statin prescription over time for the study cohort. The colored bars show the percentage of patients with at least one statin prescription during each 2-year period stratified by age category **(A)** and race **(B)**.

Statin prescriptions increased over our study period (BC diagnosis year 2008–2009: 65%, 2010–2011: 67%, 2012–2013: 66%, 2014–2015: 69%, 2016–2017: 70%; *p* = 0.01) (**Figure 1**). This trend was generally observed in all age categories except for patients 80 years and older. Patients who identified as White and other race had increased statin prescription frequencies over time. However, statin prescriptions for Black and Latina patients did not consistently increase over time, differing from the overall trend (**Figure 1**).

**Figure 2:**
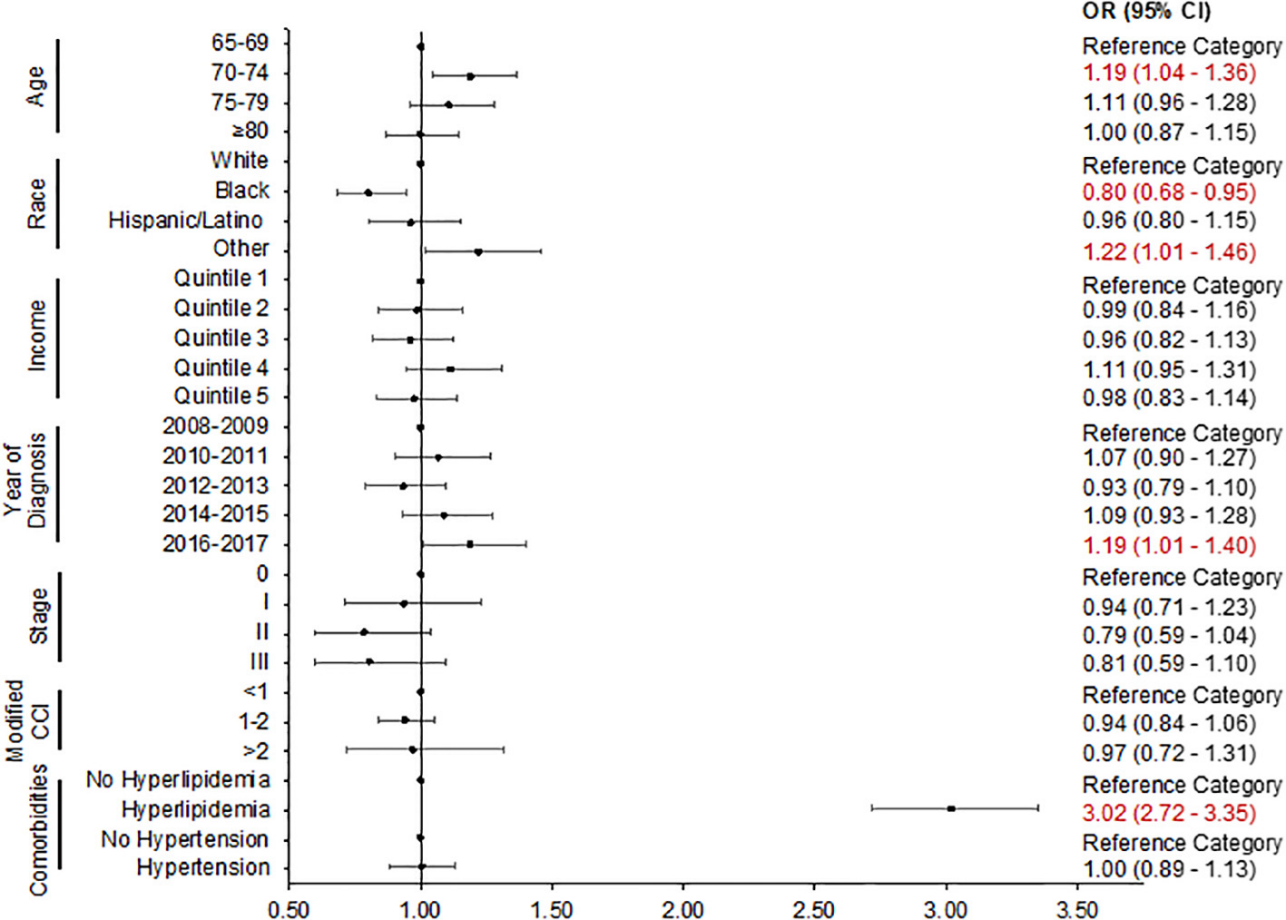
Logistic regression plot: predictors of statin prescription. The adjusted odds ratio with 95% confidence intervals is presented. CCI, Charlson comorbidity index; CI, confidence interval; OR, odds ratio.

In adjusted logistic regression (**Figure 2**), more recent BC diagnosis [2016–2017 vs. 2008–2009: odds ratio (OR): 1.19, 95% confidence interval (CI): 1.01–1.40], age group (70–74 vs. 66–69: OR: 1.19, 95% CI: 1.04–1.36), other race (OR: 1.22, 95% CI: 1.01–1.46 vs. White), and comorbid hyperlipidemia (OR: 3.02, 95% CI: 2.72–3.35) remained predictors of statin prescription. Black patients (OR: 0.80, 95% CI: 0.68–0.95 vs. White) were significantly less likely to receive a statin prescription.

## Discussion

In this nationally representative analysis of older early-stage BC patients with diabetes, statin prescription frequency for primary CVD prevention was 68% and increased between 2008 and 2017, but not for all age groups and races. As expected, the statin prescription rate in an older cohort with higher comorbidity burden was higher than what has been reported for a younger, healthier population with diabetes from 2015 to 2018 (55%) (16). Given the increased CVD risk of BC survivors with diabetes relative to survivors without diabetes, guideline-concordant statin prescription is critical in this population. This work highlights racial disparities in statin prescriptions for BC survivors. Our study reports lower prescription frequency in Black patients, which is consistent with racial disparities in statin use seen in the general diabetes population (16). Potential reasons for disparate statin prescriptions in Black patients include differences in patient beliefs regarding statin effectiveness, healthcare provider, mistrust, and financial barriers (18). Each of these presents critical opportunities for intervention and further research. Black BC survivors are at higher risk for CVD mortality than White BC survivors, especially within the first few years of diagnosis, highlighting the need for timely initiation of statins as investigated in this study (19). Elucidating statin prescription gaps for BC survivors is important for improving guideline-concordant preventive care and reducing BC survivorship disparities. Our analysis showed that statin use did not increase over time for patients ages 80 and older, reflecting a lack of clear benefit in this age group (8).

Although this study broadened the scope of previous literature on racial disparities in statin prescription, we recognize several limitations with this retrospective claims-based analysis. Comorbidities (e.g., diabetes, hyperlipidemia, and hypertension) and statin prescriptions were ascertained by insurance claims codes, which are subject to inaccuracies due to variation in healthcare provider billing practices. However, we used previously validated claims codes to enhance the accuracy of our findings. While claims codes accurately capture statin prescription patterns, they do not reflect statin utilization and adherence. Therefore, we cannot ascertain if statins were being taken as prescribed. We were not able to collect relevant biometrics, such as blood pressure and lipid levels. Therefore, we could not calculate 10-year atherosclerotic CVD risk and could not quantify what proportion of the study population truly qualified for a statin. However, we do know that >85% of patients with diabetes after 2013 are statin-eligible according to clinical guidelines (8). Another limitation with the SEER cancer registry is that race classifications rely largely on self-report, which can lead to incomplete race data and race misclassification. However, the SEER cancer registry has been shown to have complete and accurate race/ethnicity data compared to other large datasets (20). In future studies, with the increasing availability of genetic data, self-reported race/ethnicity data can be linked to genetic ancestry for a more complete picture of race/ethnicity. As this was a retrospective analysis, residual confounding may impact some of the associations that were identified. Despite these limitations, SEER-Medicare is one of the largest and most diverse validated databases, drawing valuable attention to nationwide trends and gaps in medical practice.

In summary, in a nationally representative cohort analysis of older patients with early-stage BC, statin prescriptions increased between 2008 and 2017 although there were racial disparities in statin prescription patterns. Further studies are needed to investigate the reasons for statin prescription disparities in patients with BC and diabetes and the impact of statin use on BC survivorship outcomes.

## Data Availability

Data used for this study are from SEER-Medicare, which is managed by the Centers for Medicare and Medicaid Services. While personal identifiers for medical providers and patients in the dataset have been removed, the data use agreement specifies that data cannot be shared publicly given the risk of re-identification (due to the large amount of available data). Data access can be facilitated, with appropriate approvals and data use agreement, from the Health Care Delivery Research Program at the National Cancer Institute. Requests to access these datasets should be directed to http://appliedresearch.cancer.gov/seermedicare/obtain/requests.html.
